# A pilot study of twice-weekly group-based written exposure therapy for veterans in residential substance use treatment: effects on PTSD and depressive symptoms

**DOI:** 10.1186/s13722-024-00531-0

**Published:** 2025-02-10

**Authors:** Natalia Van Doren, Fang-Hsi Chang, Amanda Nguyen, Kevin R. McKenna, Derek D. Satre, Shannon Wiltsey-Stirman

**Affiliations:** 1 Department of Psychiatry and Behavioral Sciences, Weill Institute for Neurosciences, University of California, 675 18th Street, San Francisco, CA 94107 USA; 2 Division of Research, Kaiser Permanente Northern California, Pleasanton, CA USA; 3 Department of Psychology, National Taiwan University, Taipei City, Taiwan; 4 Department of Psychology, University of California, Berkeley, CA USA; 5 VA Palo Alto Healthcare System, Palo Alto, CA USA; 6 National Center for PTSD Dissemination and Training Division, Menlo Park, CA USA; 7 Department of Psychiatry and Behavioral Sciences, Stanford University, Palo Alto, CA USA

## Abstract

**Background:**

Posttraumatic stress disorder (PTSD) is highly comorbid with substance use disorders (SUDs), resulting in high prevalence of PTSD among individuals in residential SUD care. However, there is limited research on integrating trauma treatment into residential SUD care settings. The aim of the present project was to conduct an initial evaluation of the effects of group-based Written Exposure Therapy (WET) on PTSD and depressive symptoms that was integrated into programming for individuals in residential SUD treatment.

**Methods:**

Participants were 48 Veterans with comorbid PTSD-SUD from a 28 day residential SUD program at a Veterans Affairs Medical Center. Eligible participants were enrolled in 5 sessions of WET, delivered twice-weekly in an adapted group format. PTSD symptoms and depressive symptoms were assessed at each session with the Posttraumatic Stress Disorder Checklist, DSM-5 version (PCL-5) and the Patient Health Questionnaire (PHQ-9).

**Results:**

Over 5 months, 76.2% of the target population were successfully enrolled. Of the enrolled sample, 48 participants, 92% (*n* = 44) completed 3 sessions, while 56% (*n* = 28) completed 5 sessions. Generalized Estimating Equations (GEE) showed significant within-person reductions in PTSD symptoms over time, with an average decrease of 3.18 per session (χ² = 23.21, *p* = .006) and moderate effect sizes (*d* = 0.46 and *d* = 0.51 at mid- and post-treatment). In addition, there were significant reductions in depressive symptoms within-persons over time, with an average per-session reduction of 1.13 (χ² = 23.10, *p* = .006).

**Conclusion:**

Findings demonstrate that brief, group-delivered WET is feasible and shows promise for addressing PTSD and depressive symptoms in residential SUD treatment. Results of the present evaluation could inform further efficacy testing and implementation of PTSD treatment into residential SUD settings.

**Supplementary Information:**

The online version contains supplementary material available at 10.1186/s13722-024-00531-0.

## Introduction

Posttraumatic stress disorder (PTSD) is highly comorbid with substance use disorders (SUDs). PTSD prevalence in individuals with SUD is about 30–40% in civilian samples (Back et al. [2]; Gielen et al. [12]; Kessler et al. [18]) and between 50 and 63% in Veteran samples (Roberts et al. [36]; Seal et al. [40]). Up to half of individuals in residential care for SUDs have PTSD [33], which is linked to greater impairment, homelessness, and worse psychosocial functioning (Norman et al. [29]; Riggs et al. [34]; Simpson et al. [41]). Individuals with PTSD-SUD have higher rates of return to use compared to those with either disorder alone (Bradizza et al. [8]; Killeen et al. [19]), the likelihood of which is linked to the severity of PTSD symptoms (Syan et al. [52]). Thus, addressing PTSD in people with SUD is important to facilitate long-term recovery from SUD.

Despite the high prevalence of PTSD-SUD, few residential SUD programs offer trauma treatments, with only 16.6% of US facilities and 25% of residential VA SUD facilities offering PTSD treatment (Haller et al. [13]; Spivak et al. [49]). Barriers include time constraints within a residential setting (Henslee et al. [14]); lack of clinician training and knowledge in PTSD treatments (Back et al. [3]; Killeen et al. [19]), and staffing shortages (Im et al. [17]). Moreover, current sequential treatment models (e.g., referral to PTSD treatment at discharge) pose challenges, such as long wait times and potential for worsening symptoms, which diminish the likelihood of successful engagement in follow-up care and increase likelihood of return to use (Hildebrand et al. [15]; Stimmel et al. [50]). Importantly, research supports the effectiveness of simultaneous PTSD and SUD treatment (Roberts et al. [36]). Therefore, integrated treatment models that address PTSD and SUD simultaneously present a more feasible and effective approach, improving treatment outcomes and reducing the need for readmission.

A major limitation within the existing literature is that the majority of prior work on combined PTSD-SUD intervention has been conducted in outpatient settings. Thus, there is a significant gap in our understanding of integrating PTSD treatment into SUD residential settings specifically. To date, only two randomized controlled trials have been conducted on trauma treatment in residential SUD care. Coffey et al. ([10]) found that randomization to prolonged exposure therapy (PE) resulted in greater reduction in PTSD symptoms compared to controls residential SUD patients with PTSD. However, the study excluded individuals receiving alcohol use disorder medications (e.g., naltrexone, disulfuram), limiting generalizability. In addition, Back et al. [4] found that randomization to twelve 90 min individual PE sessions was effective at reducing both PTSD and SUD symptoms in Veterans. While effective, delivering individual 90 min PE sessions for all patients with PTSD-SUD may not be feasible in most residential SUD settings, due to the high prevalence and low staffing ratios (Im et al. [17]). Thus, there is a need for further research examining PTSD treatments, particularly brief treatments that are effective and scalable, in residential SUD care.

Written Exposure therapy (WET) is a brief trauma intervention that leverages traditional exposure techniques in a written format (Sloan and Marx [43]). WET follows a 5-session sequence and primarily focuses on engaging in exposure by writing repeatedly about the index trauma event. The index trauma event is defined by the WET manual as the specific traumatic event that is identified as the most distressing or central to the individual’s symptoms of PTSD (Sloan and Marx [43]). Unlike other exposure-based treatments (e.g., prolonged exposure; cognitive processing therapy), WET does not include homework assignments and entails minimal discussion of the index trauma. Accordingly, WET may have several implementation advantages compared to other interventions, such as its brief format, the ability to conduct the therapy in a group setting while retaining confidentiality, and relatively low patient and provider burden, making it a promising approach for residential SUD settings.

A growing number of studies have been published on WET, primarily on individually delivered formats (Sloan & Marx [48]). Evidence suggests that WET is effective (Sloan et al. [46]) and has demonstrated non-inferiority to two well-established PTSD treatment models: cognitive processing therapy (Sloan et al. [47]) and prolonged exposure therapy (Sloan et al. [47]). In addition, WET has shown promise for addressing PTSD in patients and settings that face challenges in terms of time limitations. For example, WET evinced significant reductions in both PTSD and depressive symptoms in college students and fit well within their busy schedules (Morissette et al. [28]). One prior study evaluated the acceptability, feasibility, and initial effects of WET for residential SUD treatment (Schacht et al. [37]) and found that WET was feasible and acceptable, and resulted in significant reductions in PTSD symptoms. However, the study examined individually delivered WET, and it is not known whether group-delivered WET would have similar effects.

Determining effects of group-based WET is important given the majority of psychotherapy treatment in residential SUD takes place in groups (Wendt and Gone [55]). One case study examined bi-weekly group WET for individuals in residential SUD treatment (Schumacher et al. [39]) and found that the intervention was associated with PTSD symptom reduction in all three cases. However, no studies to date have examined the impact of group-based WET on PTSD symptom reduction in residential SUD programs for Veterans with PTSD. Veterans have higher PTSD symptom severity, higher likelihood of having experienced multiple traumas, and greater rates of other mental health comorbidities compared to civilians (Wisco et al. [58]). Thus, examining whether WET results in PTSD symptom reduction in this complex patient population is needed to establish initial effects before commencing an efficacy study.

We conducted a single-arm, uncontrolled pilot evaluation of group WET in a residential SUD program to examine feasibility and initial outcomes. The aim of the present project is to report on our evaluation of the effects of twice-weekly group-delivered WET on PTSD and depressive symptoms in a sample of Veterans with PTSD-SUD enrolled in a 28 day residential SUD treatment program. Groups were provided twice-weekly (as opposed to once-weekly, a more typical group therapy schedule) to increase the probability that individuals would complete the treatment within a 28 day program. We examined depressive symptoms in addition to PTSD symptoms because evidence suggests that exposure therapy for PTSD can also improve depressive symptoms (Brown et al. [9]), and that increases in negative affect and depressive symptoms predict return to use (Witkiewitz and Bowen [59]). In addition, monitoring depressive symptoms alongside PTSD symptoms is standard practice in measurement-based care for trauma-focused treatments (Fortney et al. [11]), to track outcomes comprehensively and enable enhanced safety monitoring by assessing risk for suicidality (Resick et al. [33]; Schnurr et al. [38]). Thus, we anticipated that this evaluation would provide insight into the potential impact of the WET intervention on both PTSD and depressive symptoms.

## Method

### Treatment setting

This evaluation took place in a four-week (28 day) VA residential SUD program in Northern California, which serves Veterans from California, Nevada, the Philippines, and all U.S. territories in the Pacific Basin (e.g., Guam; American Samoa). In FY 2023, 36% of patients were homeless, 28% were justice-involved, 60% were on the high-risk list for suicide, 11% had emergency room visits, 6% had a physical disability, and 16% were screened as medically complex. The standard treatment regimen on the unit includes detoxification, medications for opioid use disorder (e.g., buprenorphine), individual case management, group psychotherapy, individual psychotherapy, and psychiatric medication for co-occurring disorders. Group psychotherapy typically focuses on motivational interviewing, preventing return to substance use, cognitive behavioral therapy, and building skills to support recovery. Skills groups draw from a dialectical behavior therapy (DBT; Linehan [24]) framework, including four specific modules addressing each DBT theme—mindfulness, distress tolerance, emotion regulation, and interpersonal effectiveness. In addition, individual psychotherapy is typically tailored to meet unique needs of each patient, primarily focused on motivational interviewing and cognitive behavioral therapy to address SUD. While individual PTSD treatment (e.g., 12-session CPT or PE) is sometimes offered on a case-by-case basis for patients who extend their stay or complete an accelerated course, in the majority of cases, this is not feasible. For the participants in the present study, no PTSD treatment (evidence-based or otherwise) was offered outside of the WET group. Average length of stay in the program in FY 2023 was 28.01 days (*SD* = 15.17).

###  Participants and recruitment

WET group participants were Veterans enrolled in a 28 day residential SUD treatment program at a VA medical center between April 2023-Sept 2023. Over a five-month period, there were 82 admissions, with 63 of these patients presenting with co-occurring PTSD and SUD. Patients who had a diagnosis of PTSD at intake (assessed through clinician interviews) were invited to join the WET group by staff members. Staff confirmed interest in participation and scheduled an orientation session. Eligibility criteria for the group were: (1) sufficient memory of the index trauma to write narratives about it; (2) had approximately 2 weeks remaining at the facility (to ensure sufficient time to complete the group); (3) did not have severe suicidality and/or severe psychotic symptoms. Average length of stay before beginning WET was 10.20 days (SD = 5.21), leaving 18 days (2.5 weeks) to complete the 5-session intervention. This was often feasible due to variable discharge dates, as some patients stayed longer than 28 days or were able to extend their stay by 1–2 days to complete the WET group. While patients were eligible to be approached after 3 days in the program, typically at least a week was allotted before doing so, to allow time for detoxification and stabilization. This is guided by the Clinical Institute Withdrawal Assessment for Alcohol (CIWA; Sullivan et al. [51]) and Clinical Opiate Withdrawal Scale (COWS; Wesson et al., [56]) protocols at the treatment facility. Staff worked closely with patients to maximize attendance and coordinate with discharge dates, but due to co-occurring conditions, legal issues, or medical emergencies, some participants were discharged before completing all sessions.

## Procedures

The project was submitted to the local Institutional Review Board (IRB) and was determined to be a quality improvement project exempt from further IRB oversight, as the treatment was offered to patients as part of routine care. Eligible group participants completed a 1hour individual orientation session to discuss participation in the group, confirm eligibility and interest, and determine the index event. Patients then engaged in WET group therapy for 5 sessions. Sessions were administered twice-weekly for 1hour each. All sessions were provided by a staff member. After each session, participants completed PTSD and depressive symptom measures, as detailed below, and aligned with routine quality of care measures used in the treatment setting. The study employed a repeated measures (within-subjects) design, with PTSD and depressive symptoms assessed at multiple time points for each participant at each session.

**Orientation session:** Participants were enrolled in the residential SUD program for a minimum of 3 days before starting the WET group to allow time to acclimate and avoid acute intoxication or withdrawal effects. Eligible patients met with a staff member to orient the participant to the group procedures, provide psychoeducation about trauma treatment, and conduct a detailed assessment of trauma history, following the WET protocol (Sloan and Marx [43]). If patients reported at least one DSM-5 criterion A event, the clinician worked with the patient to identify an appropriate index trauma to be the focus of the WET group sessions. Appropriateness was determined by (1) having a clear memory of the event, (2) the event was causing significant PSTD symptoms (e.g., was the subject of PTSD flashbacks and related to significant avoidance of trauma reminders), and (3) the participants’ stated willingness to work on the event. Following the trauma assessment, staff evaluated whether patients had clinical issues that could pose a risk to themselves or others, or interfere with the effectiveness of the WET group. Concerns such as acute suicidality, aggressive or violent behavior, or active psychosis were assessed, as these could elevate the risk of iatrogenic effects, such as exacerbation of symptoms or harm to self/others. Additionally, medical conditions requiring frequent appointments were considered, as these could interfere with treatment completion. If it was determined that any of these factors posed a significant risk of harm or would prevent effective participation, the patient was deemed ineligible for the group. Finally, the staff member then provided an opportunity for the patient to ask any questions about participating along with identifying and problem-solving any roadblocks to participation (e.g., coordinating medical appointments).

**Delivery of adapted written exposure therapy (WET):** Admission to the group was on a rolling basis. Standard group size was 5 participants, with a range of 3–8. If the group included any participants attending their first treatment session, they were given psychoeducation on trauma, PTSD, the treatment rationale, and WET after completing the PCL-5. Additionally, new participants received handouts on paced breathing to help them understand the paced breathing exercise at the end of group writing sessions. The intervention was delivered by a staff member with a PhD in psychology who had prior training in WET through a combination of didactics, supervision, and consultation, consistent with the VA’s WET Evidence-Based Practice (EBP) rollout training (Worley et al. [60]), which was available to VA staff members between 2020 and 2024.

The WET intervention followed procedures outlined in Sloan and Marx ([43]), with adaptations for a group format as per Schumacher et al. ([39]). Specifically, all core elements of the original WET protocol were delivered, with the following adaptations: (1) group-based delivery format (i.e., conducting written exposures in a group instead of in individual meetings with a therapist), (2) inclusion of a brief paced breathing exercise after exposure sessions, (3) modifications to how individual feedback on trauma narratives was provided, and (4) adjustments to ensure each group member could process their writing experience.

Session 1 of WET involved psychoeducation on trauma and exposure therapy, reviewing what to expect out of treatment, and laying out the rationale for WET. Participants also learned about how to monitor distress during exposure using the verbally administered subjective units of distress (SUDS) rating scale so that they could provide distress ratings pre- and post-exposures. Following this, individuals were instructed to write for 30 min in the group about the index event, focusing on specific sensory details (e.g., what they saw, heard, smelled), as well as their thoughts and feelings about the trauma (e.g., “I was frozen with fear”) as they remembered it in the present moment. After their first exposure, they received psychoeducation on avoidance and the possibility of symptom exacerbation during treatment. After Session 1 and before Session 2, the staff clinician reviewed their written exposures and provided individualized feedback to each patient privately as part of the program’s routine check-in visits, such as whether they omitted emotions, thoughts, and sensory details, the length of the account, and whether the patient wrote about the agreed upon index trauma. In addition, safety monitoring was performed throughout by staff during and after each session by reviewing the content of written exposures to check for indications of risk (e.g., suicidal ideation), examination of PHQ-9 (Item 9) scores, and during individual check-ins as part of routine care on the unit. In Session 2, participants repeated the written exposure process from Session 1, while incorporating any feedback on the exposure process from the staff study member. In Sessions 3–5, participants receive additional instructions to write about how the trauma experience has impacted various aspects of their lives, including changes in their lifestyles, perspectives,, and relationships with others, in addition to narrating the index event as they did previously. Following 30 minutes of written exposure, the remainder of the sessions were used to allow participants to verbally reflect on the process (but not content) of the exposure experience with the group if they choose to do so. At each session, participants provided verbal SUDS ratings before and after exposure. At the end of each session, the therapist led the group in a brief 2 minute paced breathing exercise, as recommended by Schumacher et al. ([39]). This adaptation aimed to help clients manage any anxiety related to the exposure as a way to support emotion regulation and processing, especially since the group format did not allow for individual processing of acute reactions to the writing. Including these breathing techniques at the end of sessions with specific instructions not to avoid their feelings also minimized concerns about their use as safety behaviors during the exposure exercises.

**Fidelity monitoring:** To ensure treatment fidelity, the therapist followed specific guidelines from the WET treatment manual to encourage patient engagement with the trauma narrative writing process. This included providing prompts and regular encouragement during sessions to support active participation from all group members. The therapist also monitored whether patients were writing their trauma accounts by regularly reviewing them after sessions and providing feedback where appropriate. For instance, while some participants initially wrote brief accounts lacking in detail, the therapist addressed this in subsequent sessions by discussing the challenges with writing and offering additional support for expanding their narratives, as outlined in the WET manual. During the intervention, such fidelity was monitored via therapist self-checks (i.e., referring to the WET manual on a regular basis) to ensure adherence to all recommended procedures. Prior to the intervention, the therapist had was trained to fidelity in the WET via weekly supervision and consultation, consistent with the VA’s WET EBP rollout training (Worley et al. [60]).

### Measures

**PTSD diagnoses:** At admission, as is common in routine care VA settings, PTSD was assessed using electronic healthcare records (i.e., chart review) and unstructured clinical interviews performed by attending psychiatrists. Diagnoses were later confirmed by the staff clinician at the orientation session through an unstructured clinical interview.

**PTSD symptoms:** As is standard in WET, PTSD symptoms were assessed by clinical staff before each session with the validated PTSD Checklist for DSM-5 (PCL‐5 weekly version; Blevins et al. [6]) via paper-and-pen measures that were entered into the healthcare record by clinicians. The PCL-5 is a 20-item measure, with possible range 0–80, and assesses PTSD symptoms on a scale of 0 (not at all bothered) to 4 (extremely bothered). A cutoff score of 31–33 indicates probable PTSD (Bovin et al. [7]). Total scores were obtained for each patient at each session from the electronic health record. Reliability for the current sample is not available due to a lack of item-level data from patient health records.

**Depressive symptoms:** Depressive symptoms were also assessed by clinical staff before each session with the validated Patient Health Questionnaire (PHQ-9; Kroenke et al. [21]) via paper-and-pen. The PHQ-9 is a 9-item measure with possible range from 0 to 27. Scores of 0–9 indicate mild-moderate symptoms, 9–19 indicate moderate-severe symptoms, and 20–27 indicating high severity depression. Total scores were obtained for each patient at each session and entered into the healthcare record by clinicians. Reliability for the current sample is not available due to a lack of item-level data from patient health records.

### Data analysis

All analyses were conducted in *R* Version 4.1.0 and *RStudio* Version 1.4.17 (R. Core Team, [30]). Descriptive statistics were computed using the *psych* package (Revelle et al. 2023) and *tidyverse* package (Wickham et al. [57]). An intent-to-treat (ITT) approach was used to for all analyses. This included 42% (*n* = 20) of participants who did not complete all five sessions. Missing data were handled with multiple imputation by chained equations (MICE) using the *mice* package in *R* (van Buuren & Groothuis-Oudshoorn [53]). This approach generates multiple imputed datasets that were then pooled to obtain final estimates to reduce bias due to missingness. Generalized Estimating Equations (GEE) were employed to assess changes in symptoms across all five sessions, using the *geepack* package (Hojsgaard et al. [16]). The GEE analysis accounted for within-person correlations in repeated measures, using an exchangeable correlation structure to model the longitudinal data. The GEE model provided estimates of the average change in symptoms per session, with Wald’s chi-squared tests used to assess the significance of these changes. GEE uses all available data points through a population-averaged approach, making it suitable for longitudinal data analysis even when some observations are missing (Lee et al. [22]). Multiple imputation is recommended to enhance the robustness of the estimates when missing data may not meet the missing-completely-at-random (MCAR) assumption (Aloisio et al. [1]; Lipsitz et al. [25]). Accordingly, we used imputed data (as described above) to address potential biases and improve the precision of our estimates. Estimated Marginal Means (EMMs) were derived using the *emmeans* package (Lenth et al. [23]) to examine the model-based mean symptom scores at each session. To complement the GEE analysis, effect sizes for changes from Session 1 to Session 3 and Session 1 to Session 5 were calculated manually using the formula for dependent samples Cohen’s d = M_post_-M_pre_ / SD (M_difference_). All data and code are available on OSF: https://tinyurl.com/2s8585hu.

## Results

### Sample characteristics

Group participants were 48 veterans that ranged in age from 28 to 73 years (*M* = 46.29, *SD* = 13.19), and were predominantly men (87.5%). Race/ethnicity was obtained from the electronic health record as follows: White (58.3%, *n* = 28), Hispanic/Latine (22.92%, *n* = 11), Black (18.75%, *n* = 9), Asian (6.25%, *n* = 3) and other (6.25%, *n* = 3). Patients’ primary SUD diagnoses at intake (obtained from chart reviews) were as follows: Alcohol Use Disorder (79.17%; *n* = 79.17%), Stimulant Use Disorder (50%; *n* = 24), and Opioid Use Disorder (18.75%; *n* = 9). In addition, patients had a variety of comorbid mental health diagnoses other than PTSD, primarily Major Depressive Disorder (45.83%; *n* = 22), personality disorders (16.67%; *n* = 8), ADHD (14.58%; *n* = 7) and Substance-Induced Mood Disorder (10.42%; *n* = 5; (Table 1).

**Table 1:** Demographic and clinical characteristics of WET Group participants (*N* = 48)

	*N* or mean	% or SD
Age	46.29	13.19
*Gender*		
Man	42	87.50%
Woman	4	8.33%
Other	2	4.17%
*Race & ethnicity*		
White	28	58.33%
Latine	11	22.92%
Black	9	18.75%
Asian	3	6.25%
Other	3	6.25%
*Employment status*		
No employment	43	89.58%
Active employment	5	10.42%
*Mental health diagnoses*		
PTSD	48	100%
Major depressive disorder	22	45.83%
Personality disorder	8	16.67%
ADHD	7	14.58%
Substance-induced mood disorder	5	10.42%
Schizoaffective disorder	3	6.25%
Substance-induced psychotic disorder	3	6.25%
Generalized anxiety disorder	2	4.17%
Gender dysphoria	1	2.08%
Bipolar II	1	2.08%
Eating disorder	1	2.08%
*Primary SUD diagnoses*		
Alcohol use disorder	38	79.17%
Stimulant use disorder	24	50.00%
Opioid use disorder	9	18.75%

Demographics and diagnoses were based on chart review obtained from electronic health records (EHR). PTSD diagnoses were confirmed via clinician interview, as indicated in the method ADHD: attention deficit hyperactivity disorder; PTSD : post-traumatic stress disorder

## Feasibility

**Recruitment:** Over the course of five months, 48 participants were successfully recruited, representing 58.5% of the total patient population and 76.2% of those with PTSD-SUD. This demonstrates the feasibility of engaging the target population in this setting. Recruitment efforts primarily relied on direct outreach through clinical referrals, emphasizing the potential benefits of the WET group for symptom relief. Early feedback from clinicians on the unit suggested that framing the intervention as a low-burden, time-limited option was particularly effective in attracting participants who might otherwise be hesitant to engage in treatment due to concerns about time commitment. This was especially true for individuals balancing their participation with ongoing medical treatments or complex care plans, which required careful coordination across multiple providers. The recruited participants were diverse in terms of race, ethnicity, and clinical diagnoses, reflecting the broader patient population typically seen in the setting.

**Fig. 1:**
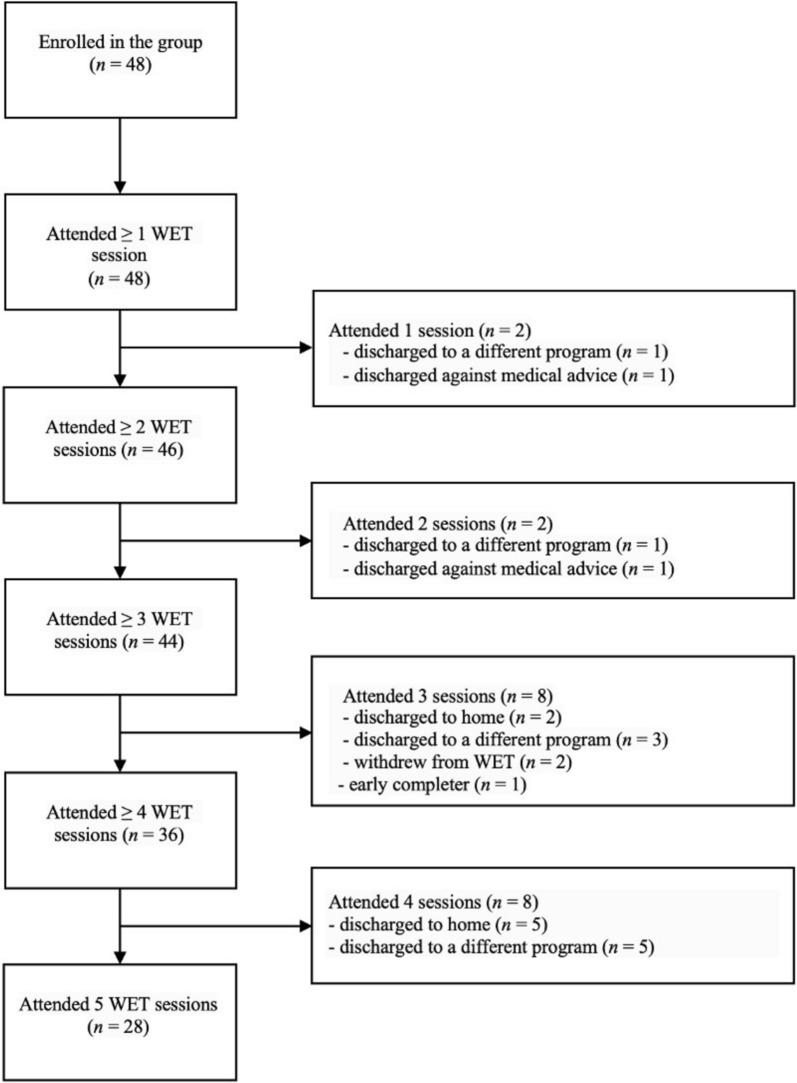
Participant flow diagram with reasons for dropout. *WET* written exposure therapy

**Retention:** Figure 1 presents a patient flow diagram indicating *n* at each session and reasons for discharge. All group participants completed at least one WET session (100%; *n* = 48). 93% (*n* = 45) completed three sessions, 75% (*n* = 36) completed four sessions, and 58% (*n* = 28) completed all five sessions. The primary reason for completing less than five sessions was discharge to home (14.5%; *n* = 7), discharge to another facility (12.5%; *n* = 6) and leaving the program against medical advice (0.06%; *n* = 3). In addition, two participants withdrew from the group due to avoidance or preference (0.04%; *n* = 2); one was an early completer (achieved symptom remission after 3 sessions and opted not to continue beyond that). Of the two participants that dropped out due to avoidance/preference, one of them decided they did not feel ready to address their trauma, while another felt the group was not helping after three sessions and decided not to continue. Demographic and clinical characteristics of completers (all 5 sessions) vs. non-completers (4 or fewer sessions) are reported in the supplement (Table S1). While there were no significant differences (Table S2), we noted a higher prevalence of unemployment amongst non-completers.

**Time Burden and Integration with Programming:** The 60 min WET sessions took place during regularly scheduled group therapy slots, specifically during art therapy and acceptance and commitment therapy (ACT). Thus, attending WET group required participants to skip the art therapy and ACT groups, but not DBT skills groups. This structure minimized disruption to their overall treatment schedule, ensuring they could attend skills groups, whereas art therapy and ACT groups are optional weekly groups. As such, WET groups were incorporated as a “separate track” for patients during those group slots. Managing ongoing medical appointments, such as hospital visits, posed a greater challenge. These appointments often required more time and were difficult to reschedule, leading to occasional missed WET sessions. However, arranging other aspects of participants’ schedules within the facility itself, such as attending DBT or other aspects of residential SUD treatment (e.g., psychiatrist and nursing appointments), was generally easier to manage and did not present significant issues for most participants. Moreover, providers reported that the group complemented other therapeutic activities, offering an intensive yet manageable intervention that did not overwhelm participants. Since the group therapy sessions took place during the time when providers would normally be delivering groups, it did not add significant time to their schedule. That said, aspects of care coordination were a bit more challenging to manage, and required additional planning to ensure participants could attend their medical appointments without missing key therapeutic interventions. Overall, the time commitment was seen as appropriate for a population balancing mental health care with other medical or life demands. This integration within the existing treatment framework suggests that the intervention’s structure was both feasible and compatible with other ongoing care.

### PTSD symptom change

**Fig. 2:**
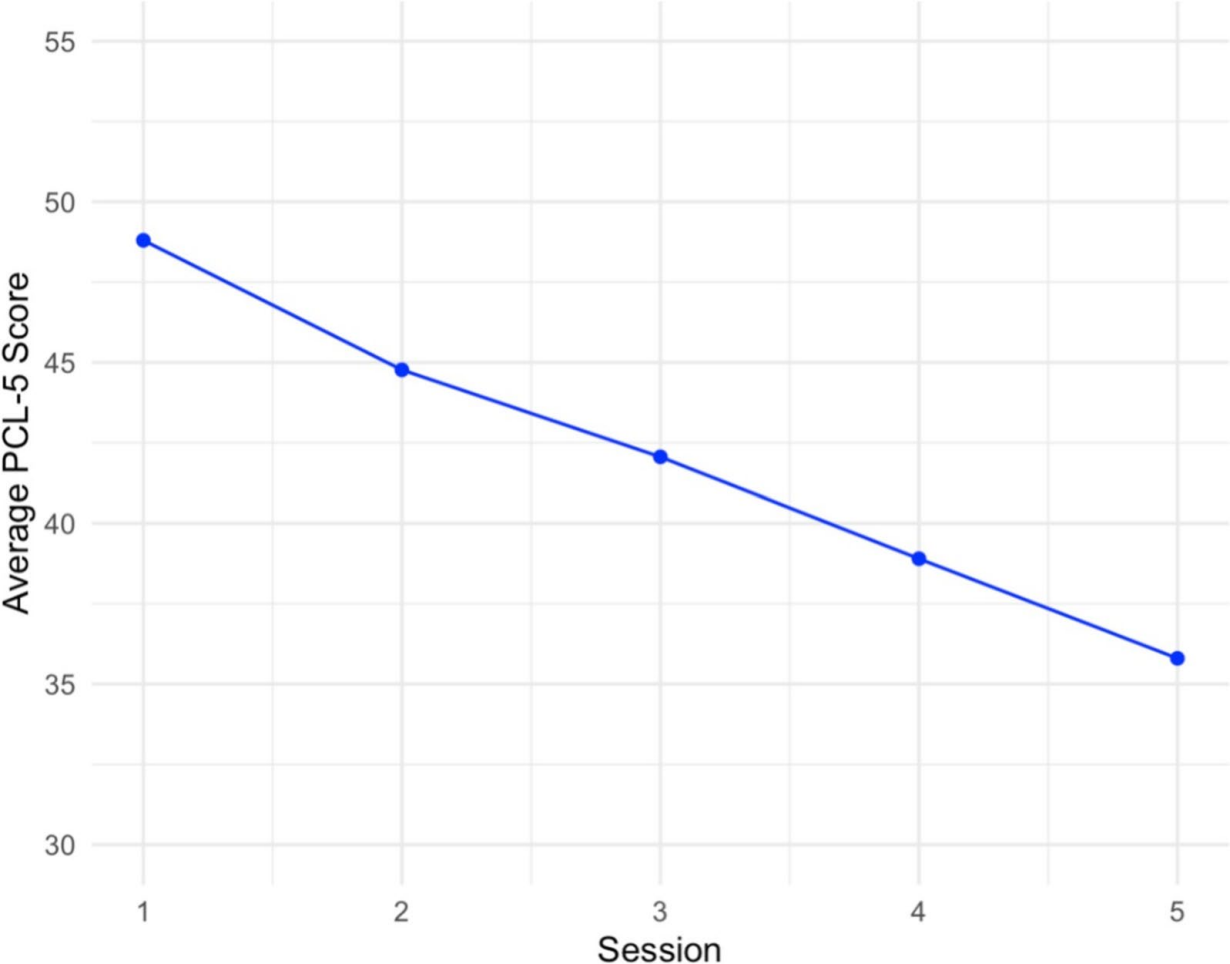
Change in PTSD symptoms over time. Average PTSD symptom scores across the entire sample from. Session 1 to Session 5. PCL-5 = PTSD Checklist, DSM-5 version.

Figure 2 depicts the average PCL-5 scores across all session in the entire sample. Results of GEE (Table 2) showed a significant reduction in PTSD symptoms over time (*b* = − 3.18, SE = 0.66, χ² = 23.21, *p* = 0.006). This suggests that, on average, PTSD symptoms decreased by approximately 3.18 points per session. Estimated Marginal Means (EMMs; Table 3) revealed the average reduction in PTSD symptoms from S1-S3 was 7.9 points, while average decrease from S1-S5 was 9.1 points. Effect sizes were moderate both S1-S3 (Cohen’s *d* = 0.46) and S1-S5 (Cohen’s *d* = 0.51). Overall, these results indicate significant within-person reductions in PTSD symptoms across all sessions, with the GEE model confirming a consistent downward trend (Fig. 2 ; Table 3).

### Depressive symptom change

**Fig. 3:**
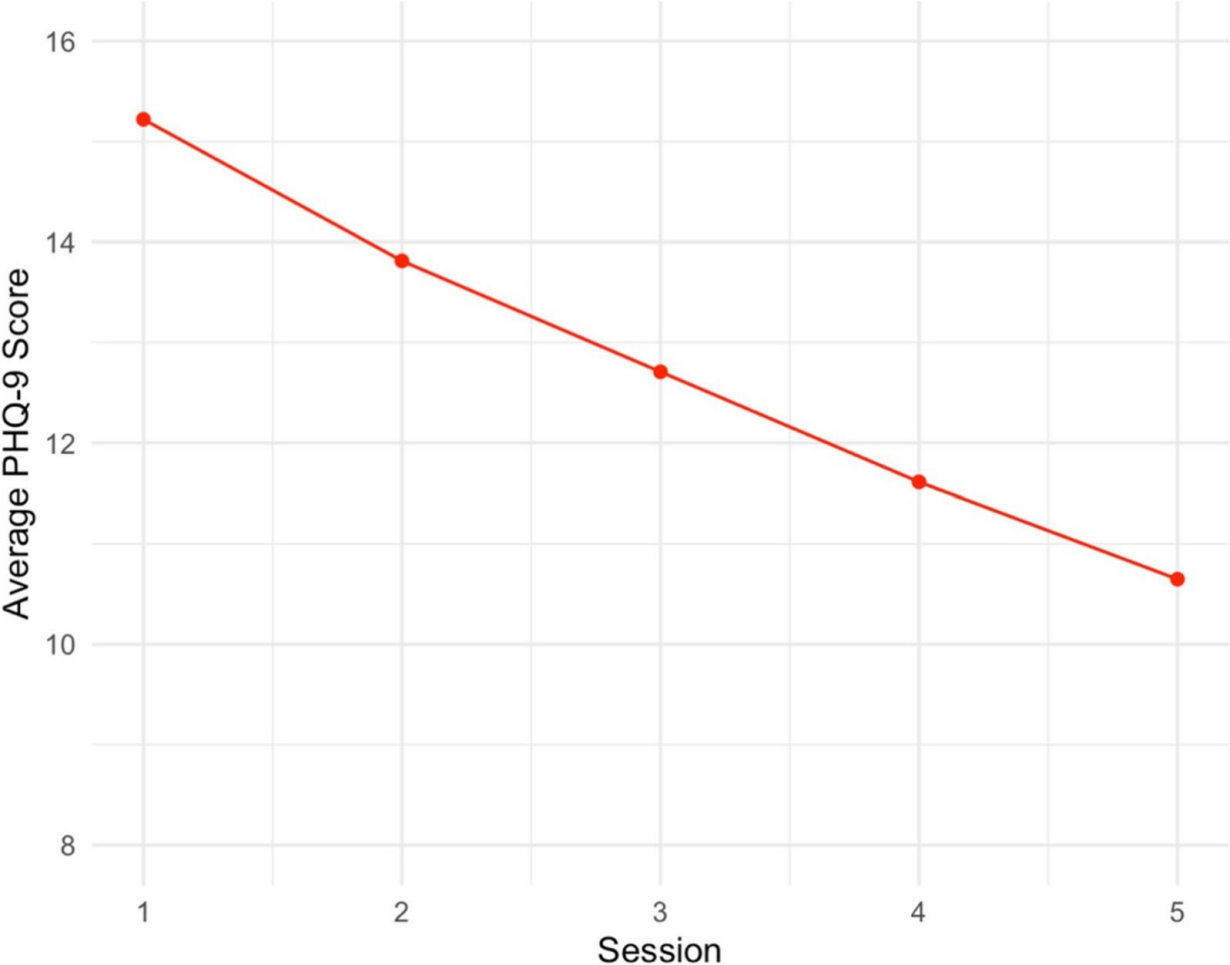
Change in depressive symptoms over time. Average depressive symptoms across the entire sample from. Session 1 to Session 5. PHQ-9 = patient health questionnaire, 9-item version.

Figure 3 depicts the average PHQ-9 scores across all sessions. Results of GEE showed significant within-person reductions in depressive symptoms over time across all five sessions (*b* = − 1.13, SE = 0.23, χ²= 23.10, *p* = .006). This suggests that, on average, depressive symptoms decreased by approximately 1.13 points per session. EMM analysis (Table 3) showed that average reduction in depression from S1-S3 was 2.8 points, with a further average decrease from S1-S5 of 2.6 points. Effect sizes were moderate for S1-S3 change (Cohen’s *d* = 0.46) and S1-S5 (Cohen’s *d* = 0.43). Overall, these results indicate significant within-person reductions in depressive symptoms across all timepoints, with the GEE model confirming a consistent downward trend (Fig. 3).

**Table 2:** Results of generalized estimating equations for repeated measures symptom change over time across all 5 sessions

Measure	GEE estimate (β)	SE	95% CI (lower, upper)	χ² (Wald)	*p*
PCL-5	− 3.18	0.66	(− 4.47, − 1.89)	23.21	0.006
PHQ-9	− 1.13	0.23	(− 1.75, − 0.51)	23.10	0.006

PCL-5: PTSD Checklist, DSM-5 version; PHQ-9: patient health questionnaire, 9-item version; GEE: generalized estimating equations; CI: confidence interval

**Table 3:** Estimated marginal means (SDs), and effect sizes for symptom change at minimal dose (Session 3) and final session (Session 5)

Measure	Session 1	Session 3	Session 5	Cohen’s d (Session 1 to 3)	Cohen’s d (Session 1 to 5)
PCL-5	48.9 (15.7)	41.0 (18.5)	39.8 (19.6)	0.46	0.51
PHQ-9	15.3 (6.34)	12.5 (6.14)	12.7 (5.9)	0.46	0.43

PCL-5: PTSD Checklist, DSM-5 version; PHQ-9: patient health questionnaire, 9-item version; SDs: standard deviations. Estimated marginal means (EMMs) were used to adjust for missing data and within-subject correlations in the repeated measures design, as recommended by a reviewer. EMMs provide a model-based estimate of the average effect at each session, accounting for the overall statistical model

### Loss of probable diagnosis of PTSD as per PCL-5 scores

Additional sub-analysis of change in PTSD symptoms amongst those who had PCL score *≥* 32 at the start of treatment to determine loss of probable diagnosis (i.e., remission) after each session (PCL-5 score < 32; Bovin et al. [7]). After two sessions, 7.7% (*n* = 3) experienced a loss of probable diagnosis after two sessions, 15.4% (*n* = 6) at Session 3, 10.3% (*n* = 4) by Session 4, and an additional 7.7% (*n* = 3) by Session 5. In total, 41.1% (*n* = 16) of participants with initial PCL-5 scores ≥ 32 experienced a loss of probable PTSD diagnosis by the end of Session 5.^1^

## Discussion

This report of a single-arm pilot evaluation represents the first investigation of the effects of WET on PTSD symptoms and depressive symptoms in Veterans undergoing residential substance use treatment. Findings suggest that twice-weekly group-delivered WET is feasible and resulted in significant within-person reductions in PTSD symptoms with moderate effect sizes in as few as three sessions, as well as after five sessions. Moreover, WET had additional benefits in terms of significant within-person reductions depressive symptoms, with small to moderate effect sizes. While causality cannot be inferred from this nonrandomized pilot, findings contribute to the growing evidence base on WET and suggest that WET is a promising approach for addressing PTSD in a comorbid SUD population in a residential setting, where time and resources may be limited to conduct a full-length exposure-based therapy protocol.

In addition to the symptom-related outcomes, the recruitment rates, retention levels, manageable time burden, successful integration with existing programming all support the feasibility of delivering WET within a residential SUD program. Specifically, enrolling 48 participants over a five-month period—representing 58.5% of the total patient population and 76.2% of those with co-occurring PTSD-SUD—demonstrates our ability to effectively engage this high-need, complex patient population. Moreover, the relatively low-burden (compared to other PTSD treatment options), time-limited nature of WET resonated particularly well with patients and providers managing other medical and psychiatric needs. Clinicians shared that this framing made the intervention appealing to participants who might otherwise avoid trauma-focused care due to time constraints or hesitations about the emotional burden.

Retention rates were promising, with the majority of participants completing a substantial portion of the intervention (58% = all five sessions, 75% = four sessions, 92% = three sessions). The primary reasons for non-completion, including discharge to another facility or to home, reflect the challenges inherent in working with a transient population that frequently transitions between care settings including outpatient treatment. Despite these barriers, the retention rates suggest that participants were able to engage with WET throughout their stay, supporting the feasibility of this intervention in residential settings. In this context, it is notable that our retention rate (58% at 5 sessions) was aligned with average rates of attrition in residential SUD programs in the US—specifically, approximately 40% of people who enroll in residential SUD treatment do not complete it (Baker et al. [5]). Further, the higher unemployment rates among non-completers suggest that structural barriers, such as the need to secure stable housing, may have impeded their ability to complete the intervention. It is notable that 80% of unemployed non-completers successfully completed at least three sessions, with 22% demonstrating loss of probable diagnosis by Session 3. These findings may suggest that a shorter, three-session WET intervention may be more feasible for unhoused or unemployed veterans, and this warrants further investigation.

The structure of the program, with WET sessions integrated into existing group therapy slots, minimized disruption to participants’ schedules and ensured that the intervention did not add undue burden to their overall treatment. The fact that participants could attend DBT skills groups and other essential substance-use related programming without missing WET sessions suggests that the intervention was well-integrated within the broader treatment framework. This is a critical consideration for residential SUD programs, which often face logistical challenges in balancing multiple therapeutic activities.

Importantly, the intervention’s brief, twice-weekly format allowed it to fit within a relatively short residential stay, which is a major advantage in a setting with limited time to complete extensive therapeutic protocols. This is particularly relevant for residential SUD programs that are often understaffed and face high turnover rates, as the shorter duration of WET may reduce the risk of attrition and allow for more participants to complete the full intervention during their stay. Moreover, the minimal additional time required for both participants and clinicians—since WET sessions replaced other group therapy sessions—further underscores the intervention’s practicality and compatibility with the existing care structure. Given the resource limitations typical of residential SUD programs, the ability to deliver a trauma-focused intervention without requiring significant additional time or staffing is a key strength of this approach. Overall, the feasibility outcomes from this pilot study provide support for the integration of WET into residential SUD programs, particularly those with limited resources and high patient turnover. Future studies could explore additional methods for formally assessing participant acceptability and clinician feedback, which would further strengthen the case for widespread implementation of WET in similar settings.

Regarding symptom reduction, participants who completed the intervention experienced significant reductions in PTSD symptoms, with an average per-session decrease of 3.18 points (overall), 7.9 points after three sessions, and 9.1 points after five sessions, respectively. These findings are consistent with and add to a growing body of work on the effects of WET for those with PTSD (Sloan & Marx, [48]) and PTSD-SUD (Schacht et al. [37]; Schumacher et al. [39]), suggesting that WET may be a feasible option for addressing PTSD in residential SUD settings. However, the observed reductions did not reach the clinically significant change threshold of a 15-point reduction on the PCL-5, as suggested by prior studies (e.g., Marx et al. [27]). It is possible that differences in treatment delivery (e.g., group-based format, shorter delivery period of 2.5 weeks rather than 5 weeks) or other factors may have contributed to the lower reductions in PCL scores in this small pilot evaluation. Moreover, the moderate effect sizes (*d* = 0.46 after three sessions, *d* = 0.51 after five sessions) may indicate room for further optimization. Tailoring WET prompts to better address the unique needs of a PTSD-SUD population—such as focusing on the specific trauma-related impacts of substance use—may help enhance outcomes in future trials. Further, while the fact that nearly half (41.1%) of participants with probable PTSD at baseline no longer met this threshold by the end of the intervention, is promising, further follow-up studies are warranted to confirm the generalizability of these results in larger samples.

Regarding depressive symptoms, we observed significant reduction across the five sessions, with an average decrease of 1.13 points per session. Although effect sizes were moderate (*d* = 0.46 after three sessions, *d* = 0.43 after five sessions), the overall reduction did not meet the clinically significant change threshold (5 scale points; Kroenke [20]). Nonetheless, observed improvements align with prior research showing that trauma-focused therapies often lead to secondary improvements in depressive symptoms (Brown et al. [9]). Future studies might explore whether participants with more severe baseline depressive symptoms benefit more, potentially allowing for greater tailoring of interventions for individuals with comorbid PTSD and depression.

Within the VA context, EBP rollouts such as WET are designed to equip providers with the skills to deliver these interventions as part of routine care and include ongoing supervision and consultation to maintain fidelity (LoSavio et al. [26]). Given this structure, our findings suggest that, with appropriate training and support, the group adaptation of WET in a SUD program is feasible and shows preliminary benefits. However, outside the VA, where WET (or other evidence-based PTSD) training may be less accessible, it is important to consider the additional resources required to implement and supervise the intervention effectively.

Overall, findings suggest that twice-weekly group-delivered WET is a promising approach for addressing PTSD treatment in PTSD-SUD populations in residential treatment settings. The fact that the intervention could be completed in as little as 2.5 weeks with twice-weekly sessions and in a group context makes it particularly appealing for implementation in residential SUD programs that are often understaffed, have high rates of attrition and turnover, and limited time to complete the intervention after assessments are performed (Im et al. [17]). Nevertheless, limitations such as the challenges of managing participants’ transitions between care settings, as well as the relatively short follow-up period, highlight the need for future research to explore strategies for improving retention and assessing longer-term outcomes.

### Strengths, limitations, and future directions

The present evaluation has several notable strengths, including examining the effects of the WET intervention in a real-world setting with the patient population for which the intervention is intended, conducting the intervention with a broad range of patients with complex comorbidities, and the use of psychometrically validated measures to assess PTSD and depressive symptoms. Moreover, findings add to the scant literature on the integration of PTSD treatment in residential SUD care. However, results should be interpreted with caution in light of several limitations. As a single-arm pilot, the lack of a control group precludes the ability to establish causal relationships between the intervention and symptom outcomes. Given that patients were undergoing treatment for SUD, it is possible that improvements occurred regardless of WET participation, such as due to other interventions they received while on the unit (e.g., substance use medications; case management). However, prior work suggests that SUD treatment alone does not by itself aid in reducing PTSD symptoms (Roberts et al. [35]; Simpson et al. [42]). Future studies should employ random assignment to establish the efficacy of WET in comparison to usual care. Assessing outcomes over a longer follow-up period would also allow investigation of whether intervention can be sustained over time.

We did not assess the effects of the intervention on other mental health outcomes (e.g., anxious symptoms) nor in relation to substance use. We opted for this approach to limit participant and clinician burden and because the aims were to establish feasibility and initial effects on PTSD primarily. Nevertheless, in future work, establishing additional benefits of the intervention on SUD-related outcomes, such as return to use, would be valuable. Moreover, assessing putative mechanisms of the WET intervention is an exciting area of future investigation—e.g., whether WET could reduce avoidance of trauma triggers.

Limits on generalizability include the small sample size, specific population (Veterans, mostly male), and setting (the specific residential SUD treatment program). Accordingly, testing the intervention not only in larger samples with random assignment, but also across other patient populations and treatment settings would be important to investigate in future work. At the same time, the target patient population and setting in this evaluation makes the work more applicable to future studies within these settings and populations. Moreover, we did not have self-reported acceptability measures from patients, an important direction for future work. Further, in this small pilot evaluation, PTSD diagnoses were assessed with chart reviews and clinician interviews, rather than the Clinician-Administered PTSD Scale for DSM-5 (CAPS-5; Weathers et al. [54]), because treatment was offered as part of routine care. Thus, results may be limited to those who received PTSD diagnoses through these methods. In addition, potential bias due to program attrition is a concern in the present evaluation; while we attempted to address this using multiple imputation and intent-to-treat analytic approach, this does not preclude the possibility missing data influenced observed outcomes. Finally, it is important to acknowledge that without a control group, regression to the mean is a potential explanation for the observed symptom improvements.

Limitations notwithstanding, findings lay the groundwork for future work integrating PTSD treatment into residential SUD programs and suggests that WET may be a promising approach for addressing PTSD-SUD in a manner that is feasible within the inpatient context. Future work with larger samples should assess effect heterogeneity (i.e., treatment moderators) to examine “what works best for whom” with respect to PTSD-SUD treatment in residential SUD programs.

## Conclusion

The present study contributes valuable initial findings regarding the benefits of brief, group-based PTSD treatment for Veterans in residential substance use treatment. Results suggest that twice-weekly group-delivered WET in residential SUD settings is feasible and may provide PTSD and depressive symptom relief in as little as three sessions. Offering WET to individuals with PTSD in residential SUD treatment programs may provide an important tool for addressing PTSD-SUD and could encourage uptake given its relatively lower provider burden compared to traditional, more extensive exposure therapy protocols. The present work sets the stage for further investigations and intervention developments to address the complex challenges of treating PTSD-SUD.

## Supplementary Information

Supplementary Material 1.

## Data Availability

All data and code are available on OSF: https://osf.io/4hkvz/?view_only=5e90af12780142ed907c1e5223a91a70.

## Footnotes

1 Sensitivity analyses using the raw data, without imputation, found a similar pattern of results, reported in the supplementary materials.
