# Supplementary material for: A pilot study of twice-weekly group-based written exposure therapy for veterans in residential substance use treatment: effects on PTSD and depressive symptoms
Source: Addict Sci Clin Pract. 2025 Feb 10;20:11. doi: 10.1186/s13722-024-00531-0 (PMC11809123; doi:10.1186/s13722-024-00531-0)
Supplement: Supplementary file 1 — Supplementary Material 1. [file 13722_2024_531_MOESM1_ESM.docx]

| **Table S1.** *Demographic and Clinical Characteristics of WET Group Participants Completed 5 Sessions (N=28) and WET Group Participants Completed Less Than 5 Sessions (N=20).* | | | | | | | |  |
| --- | --- | --- | --- | --- | --- | --- | --- | --- |
|  | Complete 5 sessions | | |  | | Complete less than 5 sessions | | |
|  | n or Mean | % or *SD* |  | | n or Mean | | % or *SD* |  |
| Age | 44.14 | 13.46 |  | | 49.3 | | 12.52 |  |
| **Gender** |  |  |  | |  | |  |  |
| Male | 25 | 89.29 |  | | 17 | | 85 |  |
| Female | 2 | 7.14 |  | | 2 | | 10 |  |
| Other | 1 | 3.57 |  | | 1 | | 5 |  |
| **Race & Ethnicity** |  |  |  | |  | |  |  |
| White | 16 | 57.14 |  | | 12 | | 60 |  |
| Latine | 6 | 21.43 |  | | 5 | | 25 |  |
| Black | 6 | 21.43 |  | | 3 | | 15 |  |
| Asian | 1 | 3.57 |  | | 2 | | 10 |  |
| Other | 1 | 3.57 |  | | 2 | | 10 |  |
| **Employment Status** |  |  |  | |  | |  |  |
| No employment | 23 | 82.14 |  | | 20 | | 100 |  |
| Active Employment | 5 | 17.86 |  | | - | | - |  |
| **Baseline Symptom Scores** |  |  |  | |  | |  |  |
| Baseline PCL-5 Score | 52.18 | 15.21 |  | | 44.25 | | 15.48 |  |
| Baseline PHQ-9 Score | 16.07 | 6.57 |  | | 14.25 | | 6.02 |  |
| **Diagnoses** |  |  |  | |  | |  |  |
| PTSD | 28 | 100 |  | | 20 | | 100 |  |
| Depressive disorder | 14 | 50 |  | | 8 | | 40 |  |
| Personality disorder | 6 | 21.43 |  | | 2 | | 10 |  |
| ADHD | 6 | 21.43 |  | | 1 | | 5 |  |
| SIPD | 2 | 7.14 |  | | 1 | | 5 |  |
| SIMD | 2 | 7.14 |  | | 3 | | 15 |  |
| Schizoaffective disorder | 1 | 3.57 |  | | 2 | | 10 |  |
| Eating disorder | 1 | 3.57 |  | | - | | - |  |
| Gender dysphoria | - | - |  | | 1 | | 5 |  |
| Bipolar II | - | - |  | | 1 | | 5 |  |
| GAD | - | - |  | | 2 | | 10 |  |
| **Primary SUD Diagnosis** |  |  |  | |  | |  |  |
| Alcohol | 24 | 85.71 |  | | 14 | | 70 |  |
| Stimulant | 11 | 39.29 |  | | 13 | | 65 |  |
| Tobacco | 11 | 39.29 |  | | 6 | | 30 |  |
| Cannabis | 8 | 28.57 |  | | 8 | | 40 |  |
| Opioid | 6 | 21.43 |  | | 3 | | 15 |  |
| SHA | 2 | 7.14 |  | | - | | - |  |
| Nicotine | 1 | 3.57 |  | | - | | - |  |
| Hallucinogen | - | - |  | | 1 | | 5 |  |
| *Note.* PTSD = post-traumatic stress disorder. ADHD = attention deficit hyperactivity disorder. SIPD = substance-induced psychotic disorder. SIMD = substance-induced mood disorder. GAD = generalized anxiety disorder. SUD = substance use disorder. Demographics and diagnoses were based on chart review obtained from electronic health records (EHR). PTSD diagnoses were confirmed via clinician interview, as indicated in the method. | | | | | | | |  |

| **Table S2.** *Independent Sample t-tests and Fisher's Exact Tests Examining Demographic and Clinical Differences between 5-session Completers (N = 28) and Non-completers (N = 20)* | | | | |
| --- | --- | --- | --- | --- |
| **Variable** | **t** | **df** | **p** | **Test Type** |
| Age | -1.35 | 46 | 0.18 | Independent t-test |
| Baseline PCL-5 Score | 1.77 | 46 | 0.08 | Independent t-test |
| Baseline PHQ-9 Score | 0.98 | 45 | 0.33 | Independent t-test |
| Gender |  |  | 1.00 | Fisher's Exact Test |
| Race |  |  | 0.79 | Fisher's Exact Test |
| Employment |  |  | 0.07 | Fisher's Exact Test |
| Psychiatric Diagnosis |  |  | 0.34 | Fisher's Exact Test |
| Primary SUD Diagnosis |  |  | 0.56 | Fisher's Exact Test |
| *Note.* PCL-5 = PTSD checklist, DSM-5 version. PHQ-9 = Patient health questionnaire, 9-item version. Independent t-tests were conducted on continuous variables (Age, Baseline PCL-5 Score, and PHQ-9 Score). Fisher's Exact Tests were conducted on categorical variables (Gender, Race, Employment, Diagnoses, and Primary Substance Use Disorder (SUD) Diagnosis). Fisher's exact test does not provide a test statistic (e.g., t or χ²); instead, it directly reports a p-value. It is used when sample sizes are small or when expected frequencies are low, and assumptions of the chi-square test may not be met (e.g., when cell size is 0 in one group). | | | | |

| **Table S3.** Unique remission cases by session for imputed and raw data. | | |
| --- | --- | --- |
| **Session Number** | **Imputed Data (N)** | **Raw Data (N)** |
| 2 | 3 | 3 |
| 3 | 6 | 6 |
| 4 | 4 | 3 |
| 5 | 3 | 3 |

*Note.* Imputed data results are based on 5 imputed datasets from the *mice* package in R. Results are pooled across all five datasets.
